# Community intervention programs prolong the onset of functional disability among older Japanese

**DOI:** 10.1111/ggi.14385

**Published:** 2022-04-22

**Authors:** Takafumi Yamamoto, Hiroyuki Hikichi, Katsunori Kondo, Ken Osaka, Jun Aida

**Affiliations:** ^1^ Department of Health Promotion National Institute of Public Health Saitama Japan; ^2^ Division of Public Health Kitasato University School of Medicine Sagamihara Japan; ^3^ Center for Preventive Medical Sciences Chiba University Chiba Japan; ^4^ Center for Gerontology and Social Science National Center for Geriatrics and Gerontology Obu Japan; ^5^ Department of International and Community Oral Health Tohoku University Graduate School of Dentistry Sendai Japan; ^6^ Department of Oral Health Promotion, Graduate School of Medical and Dental Sciences Tokyo Medical and Dental University Tokyo Japan

**Keywords:** ATET, community intervention, long‐term care needs, prevention, social participation

## Abstract

**Aim:**

This study examined the effects of a “community‐based center” intervention to prevent the onset of functional disability among residents in disaster‐affected areas.

**Methods:**

We used data from a prospective cohort study conducted from 2010 to 2016 in Iwanuma City, Japan. Participants were community‐dwelling independent adults aged ≥65 years. The exposure variable was the experience of using a community‐based center. The outcome variable was functional disability onset. The average treatment effect on the treated (ATET) was estimated by adjusting for possible confounders. Additional analysis stratified by sex was conducted considering the sex differences in social participation rates.

**Results:**

Among 3794 participants (mean ± SD age = 72.9 ±5.3 years, 46.0% men), 196 (5.2%) used the community‐based center, and 849 (22.4%) exhibited disability onset. Of those with functional disabilities, 40 (20.4%) used the community‐based center, while 809 (22.5%) did not. The ATET for functional disability onset with community‐based center activities across all participants were not significant (ATET: 0.51 years [95% confidence interval [CI] = −0.23; 1.27]). However, the direction of the effect of community‐based center activities differed by sex (ATET: −0.14, 95% CI = −2.59; 2.31 for men [*n* = 18], and 0.66, 95% CI = 0.18; 1.16 for women [*n* = 178]). Women exhibited a 15.63% (95% CI = 3.58; 27.68) increase in the time until functional disability onset.

**Conclusions:**

The use of community‐based centers was associated with a longer period without functional disability in women. **Geriatr Gerontol Int 2022; 22: 465–470**.

## Introduction

Social participation in older adults has been associated with positive health outcomes^1^, ^2^ and reduction of functional disability.^3^ According to a systematic review and meta‐analysis, infrequent social participation was associated with the onset of dementia,^4^ an important factor of long‐term care needs and functional disability. Other studies reported that the promotion of social participation is associated with a lower risk of depressive symptoms^5^ and falls.^6^

Because social participation is a modifiable determinant of well‐being and health among older adults,^7^ governments and healthcare professionals have provided opportunities for older residents to participate in social activities. The effectiveness of community intervention programs aimed at increasing social participation has been reported.^8^

Several municipalities in Japan set up “community‐based centers” to provide community‐based center activities for residents to increase social participation. Various programs, ranging from light exercises to arts and crafts, are conducted regularly at community‐based centers.^9^, ^10^ Two characteristics are important for making it easy for people to participate in community‐based centers. One characteristic is the location. Community‐based centers are set up in each municipality at the small‐district level, where participants can easily access, such as community centers, churches, shrines, elementary schools and parks. Another characteristic is the cost of participation. Most community‐based center activities are provided at a low cost. In 2017, 86.5% of Japanese municipalities implemented community‐based centers and offered activities.^11^ Community‐based centers are considered an effective option for conducting community‐based interventions, and this approach was introduced in the World Health Organization's monograph for aging.^12^

Supportive evidence for the community‐based centers has been reported in the municipality of Taketoyo Town. First, social participation through community‐based centers is associated with a lower incidence of functional^9^ and cognitive disabilities.^10^ The incidence of functional impairment among those participating in community‐based activities was reported to be 6.3% lower than that of non‐participants, and its risk was reduced by half for the participants.^9^ Furthermore, social participation through community‐based centers contributed to a one‐third reduction in the risk of developing dementia.^10^ In addition, older people of lower socioeconomic status may have been more likely to participate in the intervention,^13^ suggesting that community‐based center activities reduce health inequalities.

Natural disasters tend to increase health inequalities. For example, disasters have a negative effect on survivors' health through the destruction of built environments, destruction of social networks and increased economic difficulty.^14^ The protective health effects of community‐based center use may also be effective in disaster‐affected areas. However, in situations where the social determinants of health are changed forcibly and dramatically, the health effects of community‐based center use on disaster survivors remain unclear.

The present study examined whether the positive health effects of community‐based center participation reported in non‐disaster‐affected areas are also observed in disaster survivors.

## Methods

### Data source and participants

We used data from part of the Japan Gerontological Evaluation Study (JAGES). These data consisted of responses to a survey for physically and cognitively independent participants aged ≥65 years living in Iwanuma City. The baseline survey was conducted through a self‐report questionnaire by mail in August 2010. During the period 2010–2016, the information of community‐based center participation and incidence of functional impairment were collected and merged to baseline data. Seven months after the baseline survey was conducted, the Great East Japan Earthquake (GEJE) occurred on March 11, 2011. Iwanuma City is located 80 km west of the epicenter of the GEJE. After the GEJE, residents in Iwanuma City experienced changes in their build environment, including relocation. However, Iwanuma City continued to conduct community intervention through community‐based center activities. The target population was 8576, with 4957 finally participating (response rate, 57.8%). In total, 110 participants were excluded because of their invalid identification number, age and death before and immediately after the GEJE. To estimate the effectiveness of community‐based centers interventions, the effect of disaster‐related disability should be minimized. The exclusion criteria were as follows: (i) people who do not live in Iwanuma City as of 2016 (*n* = 342); (ii) people who developed functional disability by March 2012 (*n* = 675); and (iii) people receiving assistance with daily living at the time of the baseline survey (*n* = 46). Consequently, 3794 participants were included in the main analysis. We conducted stratified analysis by sex because few men attended the community‐based centers among participants. This observational study adhered to the reporting requirements of the STROBE statement.

### Measurements

#### Exposure variable

The exposure variable was the use of community‐based centers supported by the Iwanuma City government. Community‐based center activities in Iwanuma City began in 2008 as a city project. In 2016, 23 groups received subsidies from the city government and conducted 655 activities. Most programs included light physical activities, and the intervention time was about 1–3 h. Most of the programs were provided at a low cost (ranging from free of charge to 300 JPY per session). Depending on activities, the frequency ranged from once a week to several times a year. Data on study participants' use of community‐based centers between April 2010 and March 2011 were obtained from Iwanuma City.

#### Outcome variable

The outcome variable was the onset of the participant's functional disability. Functional disability has defined the certification of the long‐term care insurance system. The Iwanuma City government keeps a public record of the onset of long‐term care needs, and our outcome data were obtained from these records. Eligibility for long‐term care is based on a standardized, multi‐level assessment of physical and/or cognitive disability based on a personal interview and physician's examination.^15^ Following previous studies, receiving this certification was defined as the onset of functional disability.^9^, ^16^ In this study, the duration for the onset of the functional disability period was set from April 2012 to December 2016.

#### Covariates

Covariates were demographic (sex and age), socioeconomic (education and household income), health (instrumental activities of daily living [IADL], body mass index [BMI] and daily exercise) and behavioral variables (living alone, smoking and drinking). In addition, psychological distress, comorbidity and living area in 2010 were selected. Smoking and drinking are well‐known health risk behaviors. Living alone,^17^ psychological distress^18^ and comorbidity^19^ were risk factors for the onset of functional disability. In addition, their living areas were associated with their social participation.^20^ The disaster damage was also different depending on their living areas, and that possibly affected the onset of functional disability. Four small districts were included in the model to adjust the effects.

Age and equivalent income were used as continuous variables. Equivalent income was calculated by the square root of the number of household members. Education was categorized as either <9 years or >10 years. IADL was categorized as either no limitations (score 13) or some limitations (score ≤12). BMI was grouped into <18.5, 18.5–24.9 and ≥25. Daily exercise measured by daily walking time was grouped into <30 min, 30–59, 60–89 and ≥90 min. Smoking and alcohol drinking were categorized into two categories: yes and no. Psychological distress was calculated from the Geriatric Depression Scale score and used two categories, i.e., no (<4 points) and yes (>4 points).

### Statistical analysis

Inverse‐probability weighting (IPW) is a robust causal inference method that eliminates the effects of confounders using propensity scores. In this study, we applied IPW with the propensity score for community‐based center participation.

Based on this weight, the average treatment effects on the treated (ATET) were estimated in the survival model. The ATET was estimated using the potential outcome framework.

In the potential outcome model, Y1 is the potential outcome or counterfactual for that subject. For a participant who used a community‐based center, we observed Y1, and Y0 was the counterfactual outcome for that participant. The definitions of Y0 and Y1 are shown as (i) Y0: the outcome if participants did not use community‐based centers, and (ii) Y1: the outcome if participants used community‐based centers.

The ATET is the average effect of the treatment in the subgroup receiving treatment that estimates the mean difference (Y1 − Y0|*t* = 1). “|*t* = 1” denotes a limited effect on the treatment group. The potential‐outcome means are the means of Y1 and Y0 in the population. The ATET was estimated as the average difference in time to the onset of functional disability for the community‐based center‐participating group.

To address potential bias resulting from missing values, a multiple imputation method with 20 imputed datasets was used. All analyses were performed using Stata software (version 16.1; StataCorp LP, College Station, TX, USA), with the specific programs “stteffects.”^21^ The threshold for significance was set at *P* < 0.05, two‐tailed.

### Ethical approval

Ethical approval for the JAGES was obtained from the Ethics Committee of the National Center for Geriatrics and Gerontology (approval number: 992), Chiba University (approval number: 2493) and Tohoku University (approval number 21‐40).

## Results

**Figure 1:**
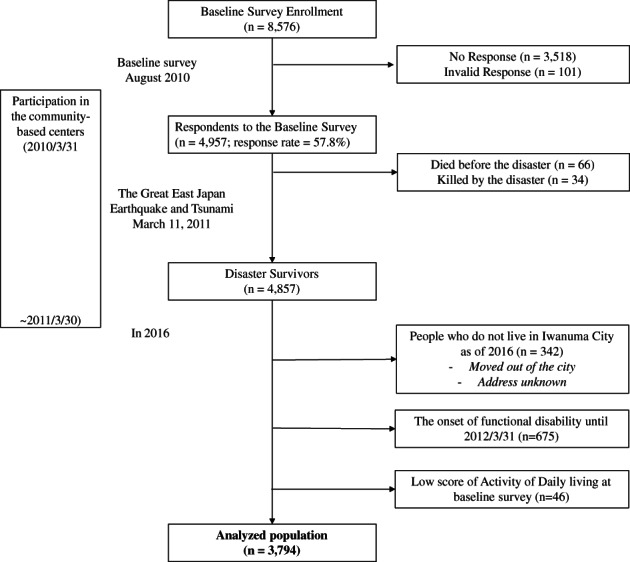
Flowchart of survey study participant.

The flowchart of the participants in this survey is shown in Figure 1. Finally, 3794 participants were included. The mean ± SD age of the participants was 72.9 ± 5.3 years, and 46.0% were men. The mean follow‐up period was 2188.3 days (range, 611–2328 days). Table 1 shows the descriptive association between baseline characteristics and the onset of disability during follow‐up. The number of participants involved in community‐based centers was 196 (5.2%), and 849 (22.4%) of all participants experienced the onset of disability. The crude prevalence rates of functional disability onset during the follow‐up of the participants who used and did not use community‐based centers were 20.4% and 22.5%, respectively. Some characteristics tended to have a higher prevalence for the onset of functional disability: women, low education, older age, living alone, low income, psychological distress, comorbidity, low exercise, low BMI and limited functional capacity.

**Table 1:** Baseline characteristics of the respondents and the proportion of the incident of functional disability

Categorical variables		Total (*N* = 3794)		Non‐participants (*N* = 3598)		Community‐based center participants (*N* = 196)	
		Disability “YES”		Disability “YES”		Disability “YES”	
		*n*	%	*n*	%	*n*	%
Sex	Men	1746	19.2	1728	19.2	18	22.2
	Women	2048	25.2	1870	25.6	178	20.2
Education (years)	≤9	1332	29.2	1239	29.5	93	25.1
	≥10	2462	18.7	2359	18.9	103	16.2
Household	Living not alone	3499	21.7	3321	21.8	178	20.1
	Living alone	295	31.1	277	31.6	18	23.6
Functional capacity	Non‐limitation	1920	16.5	1809	16.6	111	14.3
	Some limitation	1874	28.5	1789	28.5	85	28.4
Psychological distress	No	2651	20.3	2510	20.3	142	20.2
	Yes	1143	27.4	1088	27.7	54	21.1
Comorbidity	No	2687	20.4	2546	20.6	140	16.9
	Yes	1108	27.4	1052	27.3	56	29.3
Smoking	No	3356	22.7	3164	22.8	193	20.7
	Yes	438	20.1	434	20.3	3	1.6
Body mass index (kg/m^2^)	<18.5	178	33.1	168	33.9	10	19.9
	18.5–24.9	2542	21.9	2419	21.9	124	21.8
	≥25	1073	21.9	1011	22.2	62	17.8
Alcohol	No	2349	27.3	2192	27.6	157	22.3
	Yes	1446	14.5	1406	14.6	39	12.9
Daily exercise (min)	<30	1366	30	1293	30.4	74	23.1
	30–59	1366	20.4	1292	20.1	74	25.6
	60–89	544	16.9	522	17.3	22	9.1
	≥90	518	13.6	492	13.9	26	7.6
Small district	A	1046	22.2	987	22.3	59	20.3
	B	1035	19.7	1004	19.5	31	25.8
	C	1149	22.5	1109	23	40	10
	D	564	27.5	498	27.9	66	24.2

Continuous variables	Total (*N* = 3794)		Non‐participants (*N* = 3598)		Community‐based center participants (*N* = 196)	
	Mean	SD	Mean	SD	Mean	SD
Age (years)	73.2	5.9	73.2	6.0	73.1	5.4
Equivalent income (10 000 Japanese yen)	229.7	144.4	230	144.6	223.4	141.4

**Figure 2:**
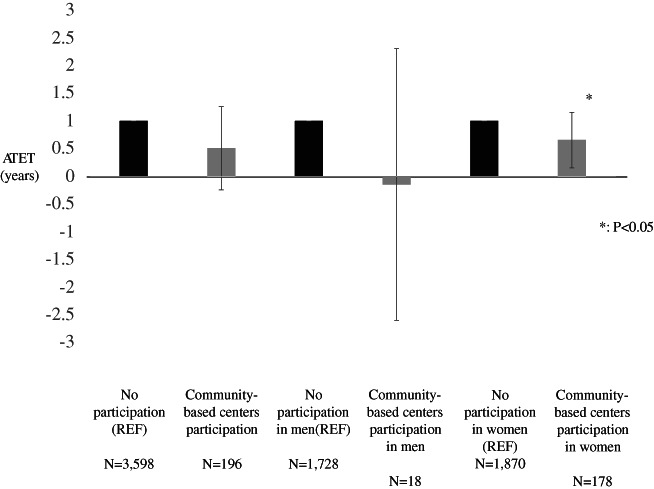
Estimation of the average treatment effects in the group that participated in the community‐based center.

Table 2 and Figure 2 show the results of the ATET estimation by community‐based center use. After adjusting for all covariates, in the participating community‐based center group, the ATET for functional disability onset with community‐based center activities was 0.51 years (95% confidence interval [95% CI] = −0.23; 1.27) shorter when they did not participate and not statistically significant (shown in Table 2 and Fig. 2). As a small proportion of men used community‐based centers, we conducted a stratified analysis by sex. For women who used the community‐based center, the ATET was 0.66 years (95% CI = 0.18; 1.16) longer than when they did not use it (shown in Fig. 2). Participation in community‐based centers prolonged the time to functional decline onset in women participants by 15.63% (95% CI = 3.58; 27.68) (Table 2).

**Table 2:** Treatment effects (ATET): change in survival time until onset of functional decline in the group that participated in the community‐based center (years)

				Stratified by sex					
				Men			Women		
		95% CI			95% CI			95% CI	
	ATET	Lower	Upper	ATET	Lower	Upper	ATET	Lower	Upper
Community‐based center participation (ref. non‐participation)	0.51	−0.23	1.27	−0.14	−2.59	2.31	0.66*	0.16	1.16
Potential‐outcome means (POmean)	4.14†	3.94	4.35	3.91†	3.45	4.38	4.22†	4.03	4.42
Effectiveness of community‐based center participation as a percentage of the total POmean estimate (%)	12.42	−5.86	30.7	−3.62	−66.22	58.97	15.63†	3.58	27.68

* *P* < 0.05, **P < 0.0001. Shows the estimated average treatment effect of community‐based center participation. Models were adjusted for all covariates: sex, age, education, equivalent income, instrumental activities of daily living, body mass index, daily exercise, smoking, drinking, living alone, psychological distress, comorbidity and living area at the baseline survey. ATET, the average treatment effect on the treated; 95% CI, 95% confidence interval.

## Discussion

The present study revealed that social participation via community‐based centers prolonged functional decline onset by 0.66 years (15.63%) among women living in disaster‐affected areas. These results are consistent with those of previous studies. Two studies of community‐based center intervention in Taketoyo Town, Japan, examined the effects of the early introduction of community‐based center interventions by the municipal government. These studies reported that the interventions reduced the risk of functional disability and dementia onset by approximately half^9^ and one‐third,^10^ respectively. Community‐based center interventions are designed to increase opportunities for social participation, and observational studies have reported their beneficial health effects, including reducing the risks of the onset of functional disability,^16^, ^22^ cognitive disability^23^ and declining activity of daily living.^24^ Community‐based centers are likely to have robust protective effects on health, regardless of whether a disaster has occurred.

There are two possible mechanisms underlying the association between community‐based center interventions and the delayed onset of functional disability. First, in Iwanuma City, most community‐based center activities were focused on exercise. Therefore, participation in community‐based centers may have increased participants' physical activity. An increase in light physical activity has a positive effect on health.^25^, ^26^ Physical activities can directly prevent or reduce disability in IADL.^27^ Second, participants formed or maintained social relationships through the use of community‐based centers. Social participation increases social networks,^2^ and social relationships have been reported to reduce the risk of cognitive decline^23^ and functional disability.^1^

The implications of our study are as follows. Community‐based centers intervention possibly reaches people in vulnerable situations, including those with lower socioeconomic status or poor health status. Many health interventions have limited effects on such populations, and this causes the “inverse care law” and the “inverse prevention law.”^28^, ^29^ However, participants in this study tended to have lower educational attainment and limitations in IADL; 45.5% of participants had a low education level, and 41.6% reported some limitations in IADL. In addition, we estimated the reduction in long‐term care costs during the follow‐up period. The per capita long‐term care cost in Iwanuma City in 2016 was approximately 1.4–2.06 million JPY, according to Iwanuma City's estimates. In this study, the functional disability onset for 36 women who participated in community‐based centers was prolonged by 0.66 years. Thus, the costs reduced during the follow‐up period were estimated to range from JPY 33 to 48.9 million. Community‐based intervention provided by local governments may help reduce health inequalities and long‐term care needs among community‐dwelling older, even in disaster‐affected areas.

This study has two strengths. First, as the ATET can reduce selection bias in the analyses of observational data, we could make robust causal inferences. Second, the follow‐up period of the present study was 6 years, which was longer compared with previous studies.^9^, ^10^ The present study confirmed that the intervention was effective for a relatively long period, even in the disaster‐affected area. Several limitations of this study should be mentioned. First, our data were obtained from one municipality. This reduces the generalizability of the results to all areas of Japan. Nevertheless, as mentioned above, the present results were consistent with those of previous studies.^9^, ^10^ Second, most of the participants who attended community‐based center activities were women. The effects of social participation could differ by sex.^30^ As a sensitivity analysis, we estimated the average treatment effect (ATE), which is valid under more severe assumptions than ATET. The ATE defines the average treatment effect in the population. The ATE results show the same trend as the ATET results (Table S1).

In the present analysis, the direction of the effect of community‐based center activity was different in sex. Several studies have been suggested that women are more likely to engage in social participation than men are.^30^ It is possible that women were more likely to participate in many of the programs offered. Consequently, the number of men involved in community‐based centers was small (*n* = 18), which may have limited our ability to address the bias. The difficulty in including men participating in community‐based center interventions is also a limitation of the public health program. Third, most of the community‐based center activities offered in Iwanuma City were centered on exercise. Therefore, there were two possibilities that the impact of community‐based center activities on the onset of functional disability was limited. First, older adults may have found it difficult to engage continuously in physical activities owing to their declining physical function. Second, healthy individuals may have been more likely to participate in community‐based center activities. Finally, due to the strong association between housing damage and functional disability, IPW could not be employed when the model included housing damage as a variable. To address this bias, the small district in which participants lived before the disaster was included in the model as a surrogate variable for housing damage.

In conclusion, community‐based center participation postponed the onset of functional disability in older women participants.

## Disclosure statement

The authors declare no conflict of interest.

## Author contributions

All authors meet all four criteria formulated by the International Committee of Medical Journal Editors regarding criteria for authorship. The study concept and design was performed by YT, AJ and OK. Data were acquired by AJ, HH and KK. The analysis and interpretation of data and critical revision of the manuscript were dealt with by all the authors. YT and AJ drafted the manuscript.

## Supporting information


**Supplementary Table S1** Treatment effects (ATET): Change in survival time until onset of functional decline (years).

## Data Availability

JAGES Iwanuma Project data used are from the JAGES study and are not third‐party data. For more information and all inquiries are to be addressed at the JAGES data management committee via e‐mail: data admin.ml@jages.net. JAGES Iwanuma Project datasets have ethical or legal restrictions for public deposition due to including sensitive information from human participants. Following Iwanuma City's regulations, which cooperated in our survey, the JAGES data management committee has imposed restrictions upon the data.
