# Supplementary material for: Community intervention programs prolong the onset of functional disability among older Japanese
Source: Geriatr Gerontol Int. 2022 Apr 22;22(6):465–70. doi: 10.1111/ggi.14385 (PMC9167703; doi:10.1111/ggi.14385)
Supplement: Supplementary file 1 — Supplementary Table S1 Treatment effects (ATET): Change in survival time until onset of functional decline (years). [file GGI-22-465-s001.docx]

Supplementary Table 1. Treatment effects (ATE): Change in survival time until onset of functional decline by community-based centers participation (years)

|  |  |  |  | Stratified by Sex | | | | | |
| --- | --- | --- | --- | --- | --- | --- | --- | --- | --- |
|  |  |  |  | Men | | | Women | | |
|  |  | 95%CI | |  | 95%CI | |  | 95%CI | |
|  | ATE | lower | upper | ATE | lower | upper | ATE | lower | upper |
| Community-based centers participation (ref nonparticipation) | -0.44 | -1.83 | 0.94 | -2.02† | -2.48 | -1.56 | 0.65† | 0.19 | 1.12 |
| Potential-outcome means (POmean) | 4.24† | 4.1 | 4.38 | 4.26† | 4.05 | 4.46 | 4.22† | 4.03 | 4.42 |
| Effectiveness of community-based centers participation as a percentage of the total POmean estimate | -10.49 | -43.08 | 22.1 | -47.46† | -57.55 | -37.37 | 15.50† | 4.17 | 26.83 |

Abbreviations: ATE, the average treatment effects 95%CI, 95% confidence interval

NOTE: This table shows the estimated average treatment effect of community-based centers participation. When all women attended the community-based centers, the ATE score was 0.66 years longer than when women did not attend the community-based centers. The rate of increased survival to the onset of functional decline was 15.63%. Models were adjusted for all covariates: sex, age, education, equivalent income, instrumental activities of daily living (IADL), body mass index (BMI), daily exercise, smoking, drinking alcohol, living alone, psychological distress, comorbidity, and living area at the baseline survey.

* P<0.05, †p<0.0001
